# Prospective nationwide analysis of long-term recurrence rates after elective ventral, incisional and parastomal hernia repairs

**DOI:** 10.1093/bjsopen/zrae070

**Published:** 2024-07-03

**Authors:** Nadia A Henriksen, Frederik Helgstrand

**Affiliations:** Department of Gastrointestinal and Hepatic Diseases, Herlev Hospital, University of Copenhagen, Herlev, Denmark; Department of Surgery, Zealand University Hospital, Koege, Denmark

Ventral and incisional hernia repairs are one of the most frequently performed elective surgical procedures in the world. Primary ventral hernias are frequent, and repairs are often performed as day case surgery with low complication rates^1^. Incisional and parastomal hernias are more complex procedures^2,3^ as previous surgical procedures and patient co-morbidity may add to the risk of complications.

Techniques for ventral and incisional hernia repair are constantly evolving including the use of minimally invasive approaches with the goal of improving outcomes^4^. A successful hernia repair is often measured by recurrence rate, although other patient-reported outcome measures may be more relevant^5^. In most studies, a 1- or 2-year recurrence rate is reported, but high-volume studies evaluating long-term follow-up are lacking.

The hypothesis for this study was that recurrence rates continue to increase over time and that recurrence rates vary substantially between types of ventral and incisional hernias. The aim was to evaluate the cumulative long-term rates of recurrence repair after primary elective umbilical, epigastric, incisional, parastomal, port-site and other ventral hernia repairs over a 15-year interval using nationwide prospectively registered data.

All ventral hernia repairs have been registered in the Danish ventral hernia database since 2007. All types of ventral hernia repairs (umbilical, epigastric, incisional, parastomal, port-site and other ventral hernias) were included from 1 January 2007 until 31 December 2022. The category ‘other ventral hernias’ comprised Spigelian, lumbar and other miscellaneous hernias of the abdominal wall. Operation for recurrence was defined as an operation for the same type of hernia registered in the Danish ventral hernia database after a primary elective hernia repair. Cumulative rate of operation for recurrence is presented in a Kaplan–Meier plot using survival analysis. Follow-up time was defined as time from index repair until the first operation for recurrence, death or last follow-up date 31 December 2022. Multivariable Cox regression analyses were performed for evaluation of variables associated with recurrence. The study was approved by the regional data protection agency of Region Zealand of Denmark (ref. REG-005-2023).

From 2007 to 2022, a total of 45 773 elective primary ventral hernia repairs were performed, of which there were 24 087 (52.6%) umbilical, 9786 (21.4%) incisional, 7654 (16.7%) epigastric, 1924 (4.2%) port-site, 1236 (2.7%) parastomal and 1086 (2.4%) other ventral hernia repairs. Median follow-up was 6.2 years (range 0–16.2).

After 15 years, operations for recurrence were 10.5% after umbilical, 14.3% after epigastric, 18–20% after incisional and port-site and 40% for parastomal hernias. The rate of operations for recurrence continued to rise throughout the study interval (*Fig. 1*). A repair without mesh and reoperation on within 90 days were factors significantly associated with risk of operation for recurrence after umbilical, epigastric, incisional and port-site hernia repair (*Fig. S1*).

**Fig. 1:**
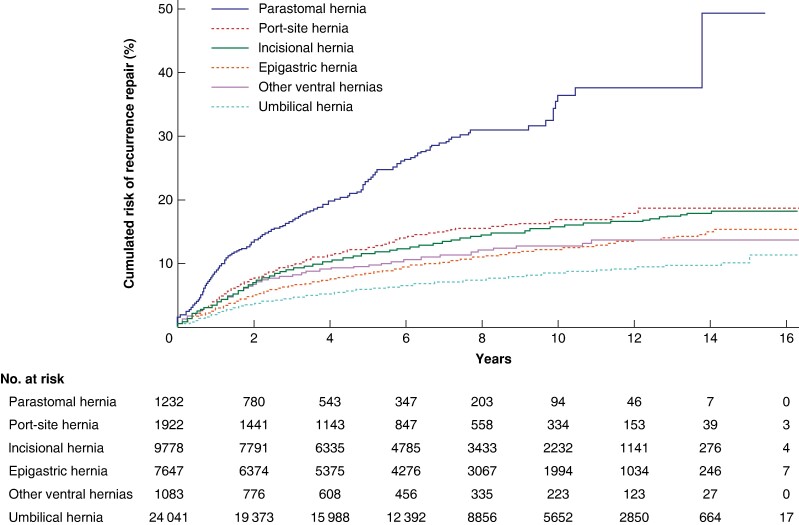
Cumulative rate of operation for recurrence after elective ventral and incisional hernia repairs from 2007 to 2022

The risk of operation for recurrence after a primary ventral or incisional hernia repair is continuously rising, even after 15 years of follow-up. The cumulative incidence of operation for recurrence was lowest after umbilical hernia repair followed by incisional and port-site hernias. Parastomal hernia repairs had 2–4 times higher long-term risk compared with all other types of ventral hernias. This equates to almost every second patient undergoing a parastomal hernia repair undergoing a further operation for recurrence.

These nationwide data suggest that longer term follow-up for patients is important as recurrence rates keep on rising. Notably, only patients operated for recurrence were included in the present study and the true recurrence rate is likely to be much higher.

## Supplementary Material

zrae070_Supplementary_Data

## Data Availability

Both authors had full access to all the data in the study and take responsibility for the integrity of the data and the accuracy of the data analysis. The data are not publicly available due to restrictions of Danish law.
