# Supplementary material for: Prospective nationwide analysis of long-term recurrence rates after elective ventral, incisional and parastomal hernia repairs
Source: BJS Open. 2024 Jul 3;8(4):zrae070. doi: 10.1093/bjsopen/zrae070 (PMC11221424; doi:10.1093/bjsopen/zrae070)

**Supplementary material**

**Figure 2.** Forrest plots of multivariable Cox regression analysis of variables associated with operation for recurrence adjusted for age, sex, and Charlson Comorbidity Index score. HR, hazard ratio


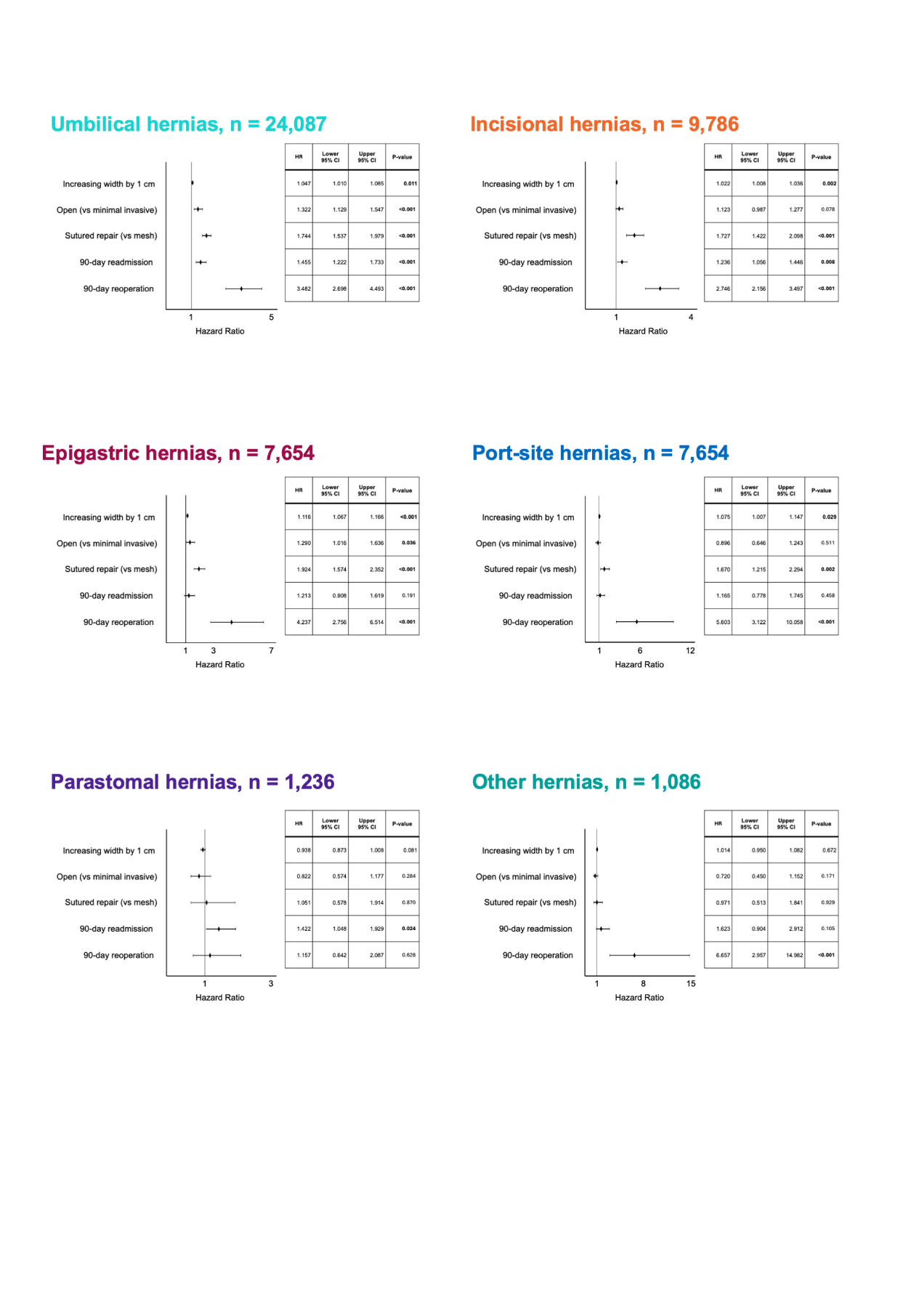

Supplement: zrae070_Supplementary_Data [file zrae070_supplementary_data.zip › Long-term-recurrence-research-letter-FINAL-210524-V3.docx]
