# Supplementary figures and images for: Prospective nationwide analysis of long-term recurrence rates after elective ventral, incisional and parastomal hernia repairs
Source: BJS Open. 2024 Jul 3;8(4):zrae070. doi: 10.1093/bjsopen/zrae070 (PMC11221424; doi:10.1093/bjsopen/zrae070)

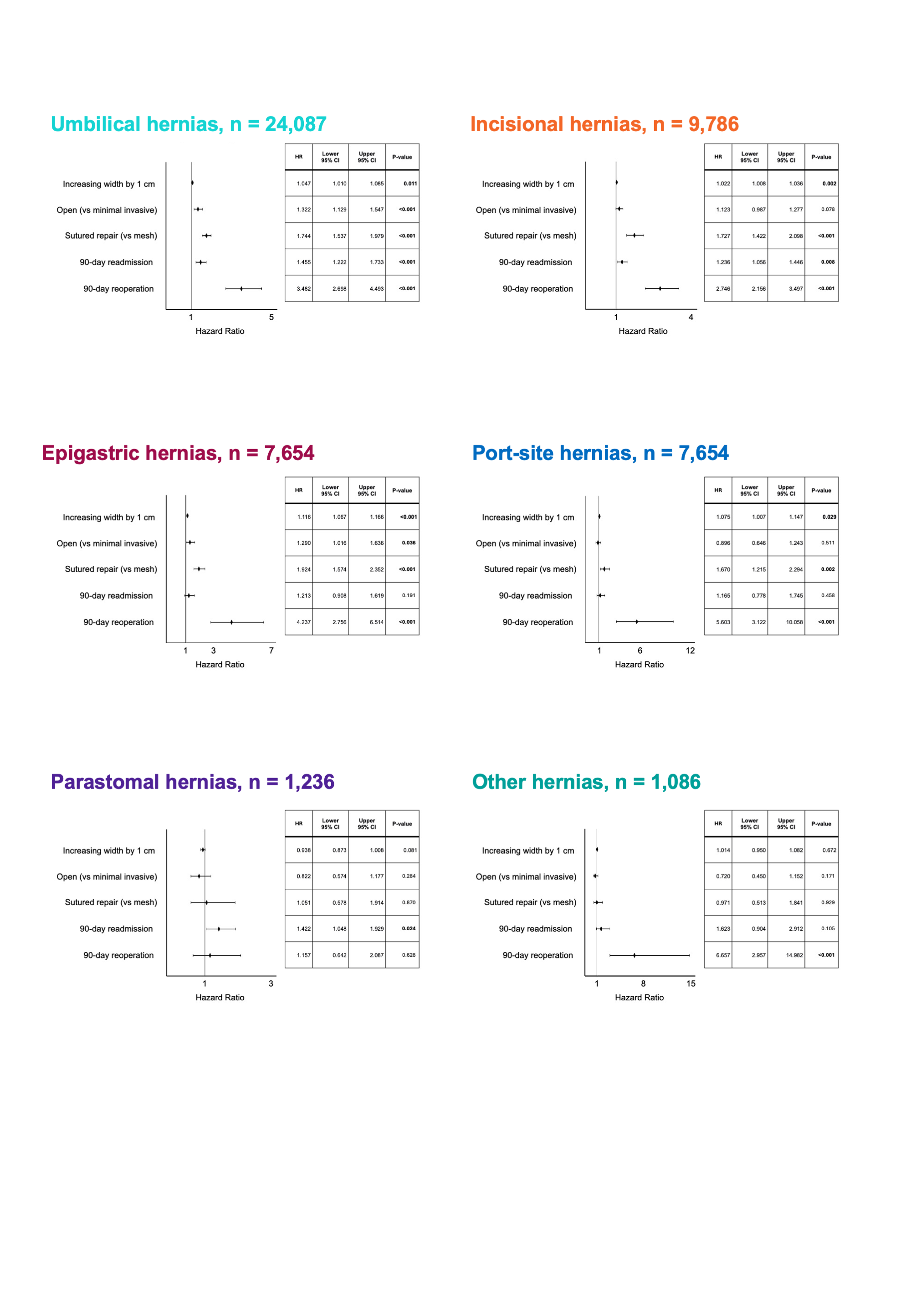

Supplement: zrae070_Supplementary_Data [file zrae070_supplementary_data.zip › Figure_2.tiff]
